# Open Access Capture of Patients With Gastroesophageal Reflux Disease Using an Online Patient-Reported Outcomes Instrument

**DOI:** 10.2196/ijmr.2101

**Published:** 2012-09-26

**Authors:** Merel M Tielemans, Jan BMJ Jansen, Martijn GH van Oijen

**Affiliations:** 1 Department of Gastroenterology and HepatologyRadboud University Medical CenterNijmegenNetherlands; 2 Department of Gastroenterology and HepatologyUniversity Medical Center UtrechtUtrechtNetherlands; 3 Department of Gastroenterology and HepatologyElkerliek HospitalHelmondNetherlands; 4 David Geffen School of MedicineDivision of Digestive DiseasesUCLALos Angeles, CAUnited States; 5 UCLA/VA Center for Outcomes Research and Education (CORE)Los Angeles, CAUnited States

**Keywords:** Gastroesophageal reflux, proton pump inhibitor, Internet, open access questionnaire, partial responsiveness

## Abstract

**Background:**

Persons with gastroesophageal reflux disease (GERD) frequently search online for information about causes and treatment options. The GerdQ self-assessment questionnaire can be used for diagnosis of GERD and follow-up of symptoms.

**Objectives:**

To assess whether it is feasible (1) to study the prevalence and impact of GERD in persons visiting a GERD information website, and (2) to identify partial responsiveness to proton pump inhibitor (PPI) therapy using the GerdQ.

**Methods:**

All visitors (aged 18–79 years) to a GERD information website between November 2008 and May 2011 were invited to complete the GerdQ online. The GerdQ questionnaire consists of 6 questions (score per question: 0–3). In respondents who did not use PPIs, we used the questionnaire to identify those with GERD (total score ≥8) and assess the influence of these symptoms on their daily life, divided into low (total score <3 on impact questions) and high impact (total score ≥3 on impact questions). In PPI users, we used the GerdQ to quantify partial responsiveness by any report of heartburn, regurgitation, sleep disturbance, or over-the-counter medication use for more than 1 day in the preceding week. We subsequently asked GerdQ respondents scoring ≥8 to complete the disease-specific Quality of Life in Reflux and Dyspepsia (QOLRAD) questionnaire.

**Results:**

A total of 131,286 visitors completed the GerdQ, of whom 80.23% (n = 105,329) did not use a PPI. Of these, we identified 67,379 respondents (63.97%) to have GERD (n = 32,935; 48.88% high impact). We invited 14,028 non-PPI users to complete the QOLRAD questionnaire, of whom 1231 (8.78%) completed the questionnaire. Mean total QOLRAD scores were 5.14 (SEM 0.04) for those with high-impact GERD and 5.77 (SEM 0.04) for those with low-impact GERD (*P *< .001). In PPI users, 22,826 of 25,957 respondents (87.94%) reported partial responsiveness. We invited 6238 PPI users to complete the QOLRAD questionnaire, of whom 599 (9.60%) completed the disease-specific quality-of-life questionnaire. Mean total QOLRAD scores were 4.62 (SEM 0.05) for partial responders and 5.88 (SEM 0.14) for adequate responders (*P *< .001).

**Conclusions:**

The GerdQ identified GERD in many website respondents and measured partial responsiveness in the majority of PPI users. Both non-PPI users with GERD and PPI users with partial responsiveness were associated with a decreased health-related quality of life. We have shown the feasibility of GERD patient identification online.

## Introduction

The Internet has gained major influence in the information supply for both physicians and patients in the last decades and has generated new opportunities to study health care and diseases [1-4]. Traditionally, medical literature, treatment guidelines, and patient brochures on gastroesophageal reflux disease (GERD) have been available mainly at the general practitioner’s office, and only 5%-30% of patients with GERD consult a general practitioner for their symptoms [5,6]. A recent study found that more than half of online health information seekers searched the Internet without prior medical consultation [4].

GERD is a chronic relapsing and remitting disorder with heartburn and regurgitation as cardinal symptoms. It is associated with a decreased health-related quality of life [7-9]. The prevalence of GERD in Western countries is 10%-20% [5,10] and the disease accounts for 3%-5% of general practitioner visits [11,12]. The main treatment focus is gastric acid suppression, for which proton pump inhibitors (PPIs) are most effective and are proven to be cost effective [13].

The majority of persons with GERD symptoms are underreported in the literature, because prior studies regarding GERD were mainly conducted in primary care [14]. Most persons with GERD symptoms do not visit a primary care physician, which is a potential limitation in the understanding of symptom prevalence and treatment response. A German study assessed gastrointestinal symptoms and quality of life via an Internet questionnaire in 5256 individuals between 2002 and 2005 [15]. This study concluded that the generated data were in general comparable with non-Internet studies, with the exception that the Internet population was younger. Since then, only a few studies have been conducted on the prevalence of a condition in the general population via the Internet. The majority of Internet-based studies invite participants by email, for example, selected by clinicians or Internet panels [16,17], thereby preselecting participants.

The aims of the current study were to assess whether it is feasible to study the prevalence and impact of GERD in persons visiting a GERD information website and to identify partial responsiveness to PPI therapy using the GerdQ self-assessment questionnaire. Symptom scores were compared with a validated health-related quality-of-life instrument. We hypothesized that the prevalence of GERD in our Internet population would be high and that a higher GerdQ score would reflect a lower health-related quality of life.

## Methods

### Study Population

The website www.maagzuur.nl contains information regarding GERD symptoms, possible causes, lifestyle advice, and treatment and diagnostic options. In May 2008, the Dutch translation of the GerdQ self-assessment questionnaire was launched on this website and could be completed by all website visitors (see Multimedia Appendix 1). After a preparatory period of 6 months, questionnaires completed between November 24, 2008 and May 4, 2011 were included in this study. We excluded respondents younger than 18 and older than 79 years. In the case of duplicate GerdQ questionnaires—defined as having an identical Internet protocol address, birth year, and gender—we included only the first completed GerdQ questionnaire. Respondents who scored ≥8 on the GerdQ were subsequently asked to complete the Quality of Life in Reflux and Dyspepsia (QOLRAD) questionnaire.

### The GerdQ Self-Assessment Questionnaire

The GerdQ is a short and validated self-assessment questionnaire that assesses presence of GERD and determines the influence of symptoms on a patient’s daily life [18]. The GerdQ comprises six questions reflecting symptoms in the previous 7 days, and has been developed with questions from the Reflux Disease Questionnaire, the Gastrointestinal Symptom Rating Scale, and the Gastrointestinal Symptom Scale, all of which are validated disease-specific questionnaires [19-21]. The GerdQ consists of the following questions referring to the previous week: (1) How often did you have a burning feeling behind your breastbone (heartburn)?, (2) How often did you have stomach contents (liquid or food) moving upward to your throat or mouth (regurgitation)?, (3) How often did you have a pain in the center of the upper stomach?, (4) How often did you have nausea?, (5) How often did you have difficulty getting a good night’s sleep because of your heartburn and/or regurgitation?, and (6) How often did you take additional medication for your heartburn and/or regurgitation, other than what the physician told you to take (such as Maalox)?

The first two questions (1 and 2) are positive predictors of GERD, where a higher symptom frequency is indicated by a higher score. Questions 3 and 4 address dyspeptic symptoms that decrease the probability of having GERD—that is, they are negative predictors of GERD. The two final questions (5 and 6) assess the impact of symptoms on a person’s daily life and are also positive predictors of GERD. The score on every question ranges from 0 to 3 for the four positive predictors of GERD (0 days is a score of 0; 1 day scores 1; 2–3 days scores 2, and 4–7 days scores 3, or in reversed order for the two negative predictors of GERD). In people who do not use a PPI, a GerdQ score of ≥8 indicates a high probability of having GERD. A cut-off of ≥3 on the GERD-impact questions 5 and 6 indicates a high impact of symptoms on a person’s daily life. We defined partial responsiveness in PPI users as more than 1 day of having heartburn (question 1), regurgitation (question 2), sleep disturbance (question 5), or over-the-counter acid suppressive medication use (question 6), all during the preceding week. We also analyzed partial responsiveness using a more stringent definition of persistence of heartburn, regurgitation, sleep disturbances, or over-the-counter medication use for at least 4 days during the preceding week. The questionnaire was shown to respondents together with a figure of a human torso with the breastbone and center of the upper stomach being marked.

### QOLRAD Questionnaire

The validated disease-specific QOLRAD questionnaire was developed to monitor health-related quality of life in patients with heartburn and dyspepsia. It contains 25 questions clustered in five domains: emotional distress, sleep disturbance, food and drink problems, physical and social functioning, and vitality [22,23]. Every question was assessed on a 7-point Likert scale, with a lower score indicating a more severe impact on daily functioning (1 = always, 2 = usually, 3 = frequently, etc, to 7 = never) [24].

### Data Analysis

Questionnaires were stored online in a specially designed website content management system (TripTic bv, Eindhoven, The Netherlands). Data were analyzed using SPSS version 16.0 (IBM Corporation, Somers, NY, USA). We calculated total GerdQ score by summing scores for all of the GerdQ questions. The mean age of respondents with high-impact GERD and low-impact GERD were analyzed using the Student *t *test. The mean age of PPI users with adequate relief and partial responders were also compared by Student *t *test. We compared dichotomous variables, such as gender, by chi-square analysis. Over-the-counter medication use and duration of symptoms were analyzed using descriptive statistics. An overall mean QOLRAD score was calculated by summing scores for all QOLRAD questions, divided by 25 for subgroups of PPI users and non-PPI users. We also calculated a mean score for each domain for respondents with high-impact GERD, low-impact GERD, PPI users with adequate relief, and partial responders to PPI therapy. In respondents with partial responsiveness, we analyzed subgroups of respondents with symptoms persisting at least 4 days per week versus those with less frequent symptoms. Using the Mann-Whitney *U *test, we compared mean scores in each QOLRAD domain between non-PPI users with low impact and those with high impact, and between PPI users with relief and those with partial response. We also compared mean scores in each QOLRAD domain between partial responders with symptoms persisting at least 4 days per week and those with symptoms persisting at most 3 days per week. A *P *value of <.05 was considered statistically significant.

## Results

**Figure 1:**
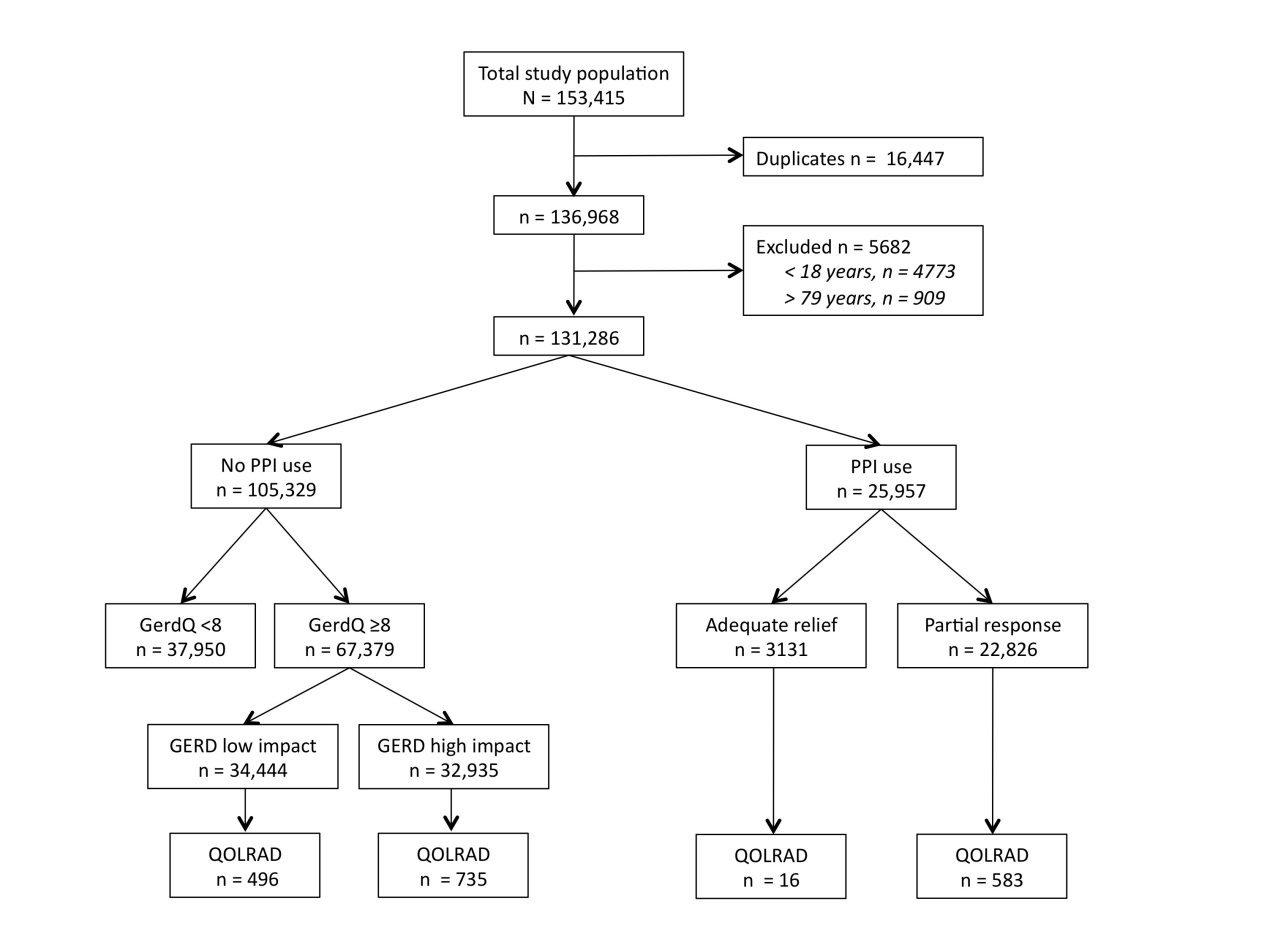
Flow of participants through the study. PPI = proton pump inhibitor. GERD = gastroesophageal reflux disease. QOLRAD = Quality of Life in Reflux and Dyspepsia.

The GerdQ self-assessment questionnaire was completed 153,415 times between November 2008 and May 2011. After removing duplicate entries (n = 16,447) and excluding respondents aged less than 18 years or 80 years and over (n = 5682), we entered 131,286 GerdQ questionnaires into our analysis (Figure 1). A total of 105,329 respondents (80.23%) reported no use of PPIs and 25,957 respondents (19.77%) reported PPI use (Figure 1).

### Respondents Without Proton Pump Inhibitor Use

The mean age of the 105,329 respondents who did not use PPIs was 41.6 (SD 14) years, and 49.72% (n = 52,369) were male. A total of 37,950 respondents (36.03%) scored <8 on the GerdQ, indicating a low probability for GERD. The remainder (n = 67,379; 64.0%) scored ≥8, of whom half (n = 32,935; 48.88%) reported GERD with a high impact on the respondent’s daily life. Respondents with GERD were older than those without GERD, and the mean age was even higher in respondents with GERD with high impact (Table 1).

**Table 1:** Baseline characteristics of respondents with and without proton pump inhibitor (PPI) use.

Characteristic		No PPI use			PPI use	
		No GERD^a^ (n = 37,950)	Low-impact GERD (n = 34,444)	High-impact GERD (n = 32,935)	Adequate relief (n= 3131)	Partial response^b^ (n = 22,826)	
Male, n (%)		17,562 (46.28%)	18,035 (52.36%)^c^	16,772 (50.92%)	1,539 (49.15%)^d^	10,132 (44.39%)	
Age (years), mean (SD)		39.2 (14)	41.7 (14)^c^	44.3 (14)	49.9 (14)^d^	48.3 (14)	
**Age categories (years), n (%)**						
	18–30	12,937 (34.09%)	9346 (27.13%)^c^	6500 (19.74%)	349 (11.15%)^d^	2719 (11.91%)
	31–40	7953 (20.96%)	7096 (20.60%)	6721 (20.41%)	437 (13.96%)	3821 (16.74%)
	41–50	8157 (21.49%)	8051 (23.37%)	8252 (25.06%)	717 (22.90%)	5787 (25.35%)
	51–60	5833 (15.37%)	6237 (18.11%)	7217 (21.91%)	861 (27.50%)	5815 (25.48%)
	61–70	2575 (6.79%)	3038 (8.82%)	3527 (10.71%)	603 (19.26%)	3644 (15.96%)
	71–79	495 (1.30%)	676 (1.96%)	718 (2.18%)	164 (5.24%)	1040 (4.56%)

^a ^Gastroesophageal reflux disease.

^b ^Partial response: heartburn, regurgitation, sleep disturbance, or over-the-counter medication use for >1 day during the preceding week.

^c^ *P *< .001 comparing low-impact GERD versus high-impact GERD.

^d^ *P *< .001 comparing adequate relief versus partial response in PPI users.

Of respondents with low-impact GERD, 61.59% (n = 21,215) took over-the-counter medication less than once per week, compared with 8.64% (n = 2846) of respondents with high-impact GERD (Table 2).

**Table 2:** Frequency of over-the-counter medication use in respondents with and without proton pump inhibitor (PPI) use.

Frequency (days/week)	No PPI use			PPI use	
	No GERD^a^ (n = 37,950)	Low-impact GERD (n = 34,444)	High-impact GERD (n = 32,935)	Adequate relief (n = 3131)	Partial response^b^ (n = 22,826)
<1	31,673 (83.46%)	21,215 (61.59%)	2846 (8.64%)	2221 (70.94%)	8352 (36.59%)
1	4086 (10.77%)	9128 (26.50%)	3169 (9.62%)	910 (29.06%)	2195 (9.62%)
2–3	1692 (4.46%)	4101 (11.91%)	13,427 (40.77%)	0 (0%)	4587 (20.10%)
4–7	499 (1.31%)	0 (0%)	13,493 (40.97%)	0 (0%)	7692 (33.70%)

^a ^Gastroesophageal reflux disease.

^b ^Partial response: heartburn, regurgitation, sleep disturbance, or over-the-counter medication use for >1 day during the preceding week.

In a subset of respondents we inquired about duration of symptoms. Of those with low-impact GERD, 45.6% (n = 554) reported symptom duration of 1 year or less, while 56.3% (n = 930) of those with high-impact GERD reported symptoms for more than 2 years (Table 3).

**Table 3:** Duration of symptoms in respondents with and without proton pump inhibitor (PPI) use.

Duration (months)	No PPI use		PPI use	
	Low-impact GERD^a^ (n = 1215)	High-impact GERD (n = 1652)	Adequate relief (n = 185)	Partial response^b^ (n = 1381)
0–6	376 (30.95%)	290 (17.55%)^c^	34 (18.4%)	190 (13.76%)^d^
7–12	178 (14.65%)	213 (12.89%)	14 (7.6%)	123 (8.91%)
13–24	130 (10.70%)	219 (13.26%)	13 (7.0%)	131 (9.49%)
>24	531 (43.70%)	930 (56.30%)	124 (67.0%)	937 (67.85%)

^a ^Gastroesophageal reflux disease.

^b ^Partial response: heartburn, regurgitation, sleep disturbance, or over-the-counter medication use for >1 day during the preceding week.

^c^ *P *< .001 comparing low-impact GERD versus high-impact GERD.

^d^ *P *= .28 comparing adequate relief versus partial response.

**Figure 2:**
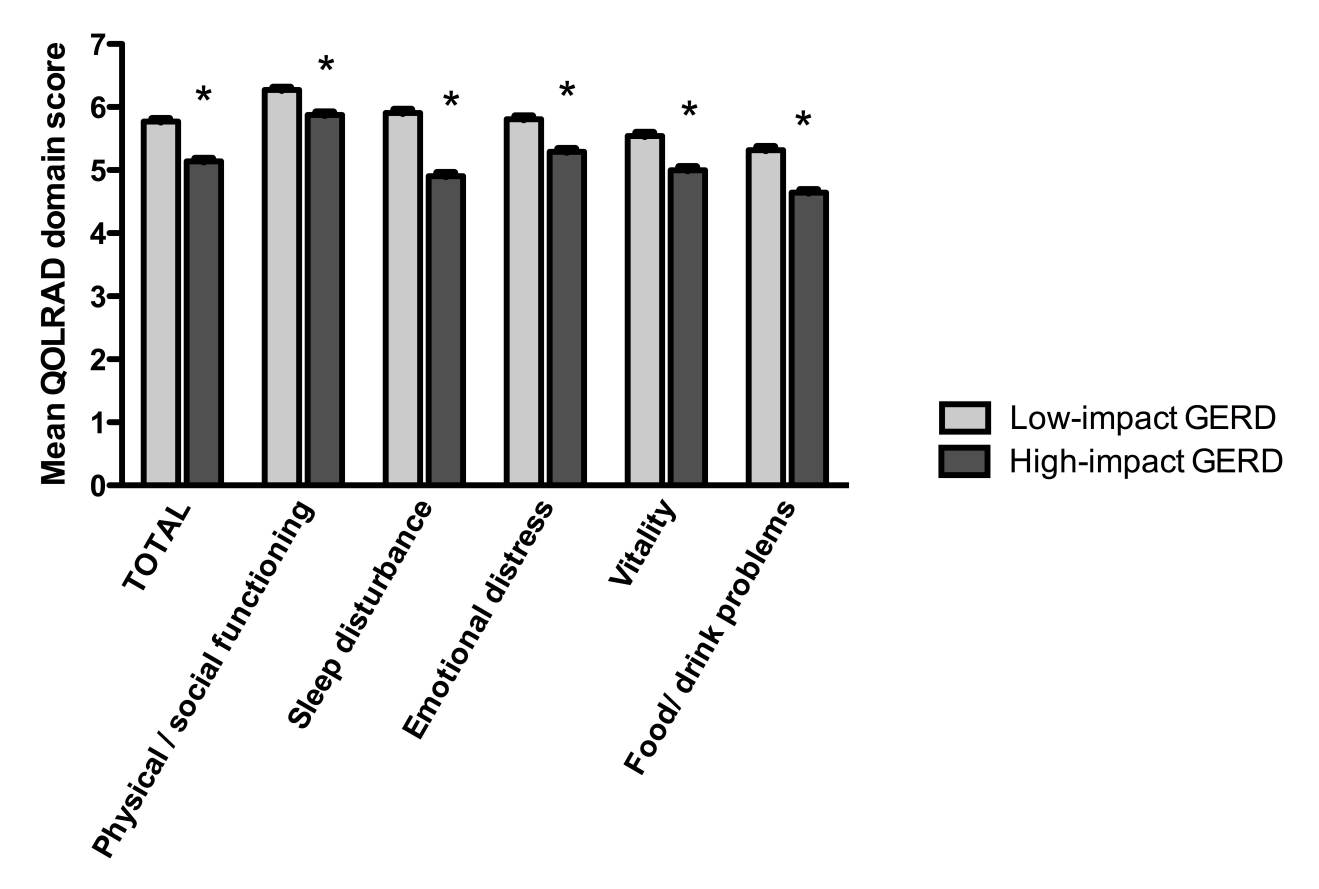
Quality of Life in Reflux and Dyspepsia (QOLRAD) scores by domain in respondents with gastroesophageal reflux disease (GERD) who did not use proton pump inhibitors (PPIs). Error bars indicate SEM. *P < .001.

A total of 14,028 respondents were eligible for (ie, GerdQ score ≥8) and invited to complete the QOLRAD questionnaire, of whom 1231 (8.78%) completed the questionnaire. The total mean QOLRAD score in respondents with GERD with low impact on daily life was 5.77 (SEM 0.04), compared with 5.14 (SEM 0.04) in those with high-impact GERD (*P *< .001; Figure 2). Quality of life was most impaired in the food/drink domain, and the differences in scores between high-impact and low-impact GERD were most pronounced in sleep disturbances and food/drink problems.

### Proton Pump Inhibitor Users

The mean age of PPI users was 48.5 (SD 14) years, and 44.96% (n = 11,671) were male. A total of 22,826 PPI users (87.94%) reported having heartburn or regurgitation, sleep disturbances due to GERD symptoms, or intake of over-the-counter acid suppressive medication for more than 1 day per week. We classified these PPI users as partial responders, and this subgroup was younger and had a higher proportion of women (Table 1). Over-the-counter medication use for at least 4 days per week was reported by 33.70% (n = 7692) of PPI users with partial response, whereas the majority of adequate responders (n = 2221, 70.94%) reported over-the-counter acid suppression medication use of less than once per week (Table 2). After applying a more stringent definition of partial response, of symptoms persisting at least 4 days per week, we obtained a total of 15,975 (61.54%) reporting partial response.

**Figure 3:**
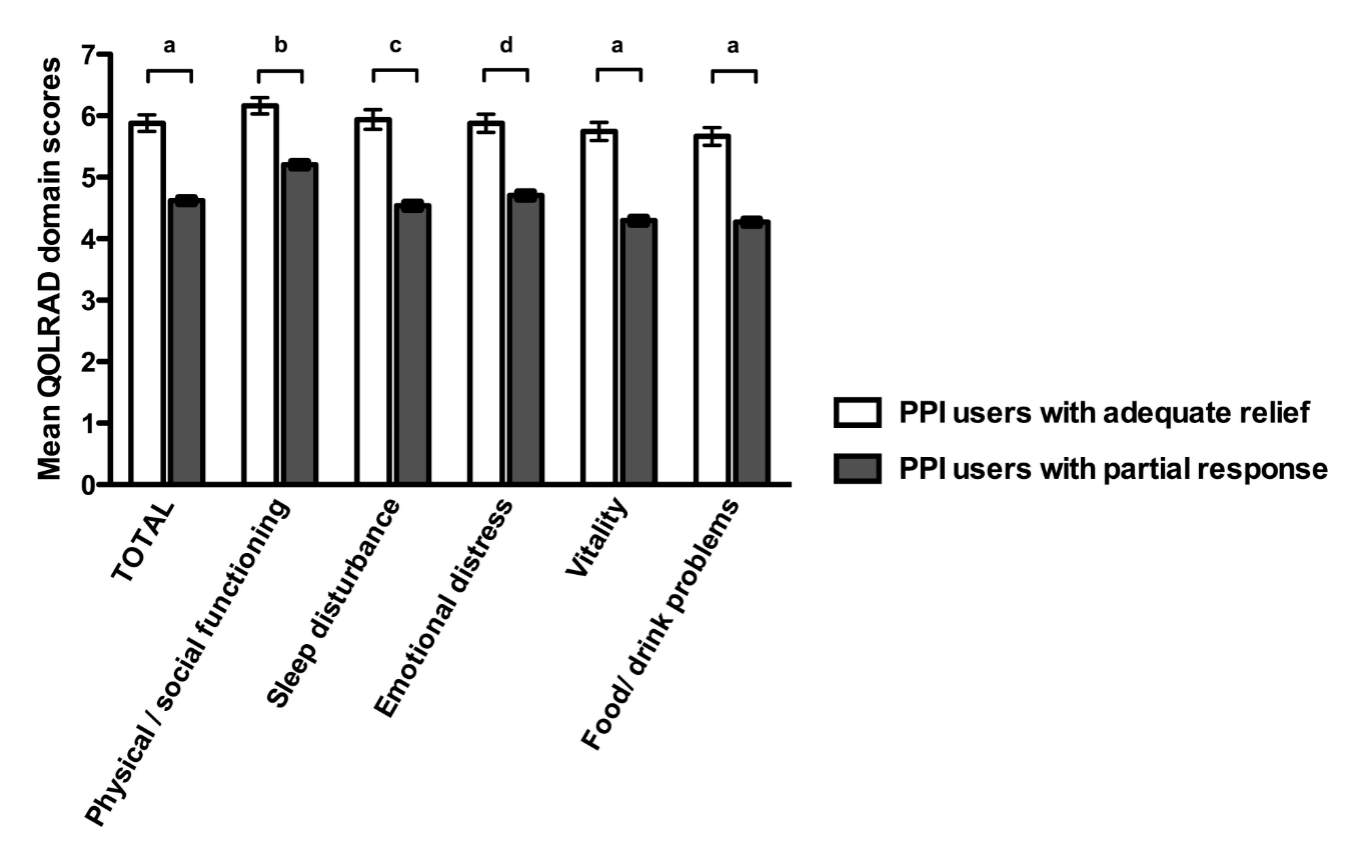
Quality of Life in Reflux and Dyspepsia (QOLRAD) scores by domain in proton pump inhibitor (PPI) users. Error bars indicate SEM. ^a^ ** < .001, ^b^ *P* = .003, ^c^ *P* = .001, ^d^ *P* =.002.

A total of 6238 PPI users were eligible for and invited to complete the QOLRAD questionnaire, of whom 599 (9.60%) completed the disease-specific quality-of-life questionnaire. The total mean QOLRAD score over all domains was 5.88 (SEM 0.14) in PPI users with adequate relief and 4.62 (SEM 0.05) in PPI users with partial response (*P *< .001; Figure 3).

**Figure 4:**
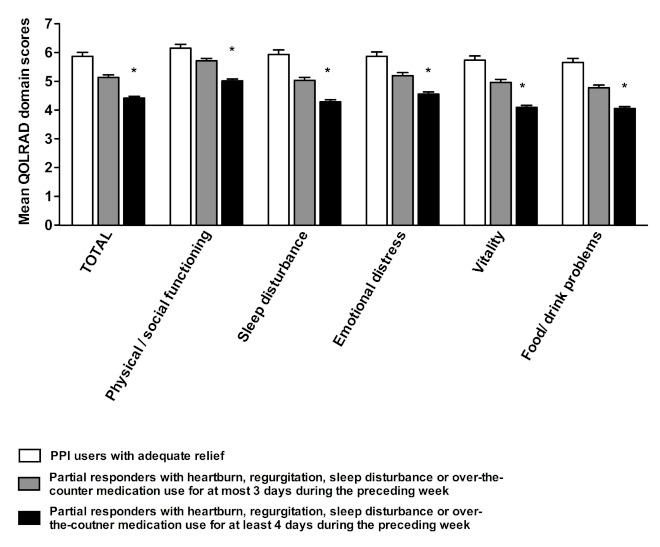
Quality of Life in Reflux and Dyspepsia (QOLRAD) scores by domain in proton pump inhibitor (PPI) users with subdivision of partial responders. *P < .001 for comparison between partial responders with symptoms persisting at most 3 days per week and those with symptoms persisting at least 4 days per weeks. Error bars indicate SEM.

In both groups of PPI users, scores in the vitality and food/drink domains were lowest, with a consistently lower score in those with partial response. The total mean QOLRAD scores in the two subgroups of partial responders were 5.14 (SEM 0.09) for responders with symptoms persisting at most 3 days per week and 4.43 (SEM 0.06) for responders with symptoms persisting at least 4 days per week (*P *< .001 for all domains; Figure 4).

## Discussion

### Principal Results

We found that the prevalence of GERD in website visitors was high, as over 60% of responders without PPI use scored at or above the predefined cut-off on the GerdQ questionnaire. Of the respondents with GERD who did not use a PPI, 49% reported that their symptoms had a great influence on their daily life, in the form of sleep disturbances, and that they needed over-the-counter medications. This was associated with a decreased health-related quality of life. Almost 90% of PPI users reported persistent GERD symptoms for at least 1 day per week. Partial responders taking PPI therapy had a lower health-related quality of life than those who did not use PPIs and those with adequate symptom relief obtained from PPI therapy.

We used the validated self-assessment questionnaire GerdQ to assess the prevalence of GERD among website visitors. Research via the Internet has several advantages and generates new possibilities. As only a minority of patients with GERD visit a health care provider, we can use the Internet to study people who are normally out of the scope of traditional research methods [25]. Another advantage is that missing answers can be directly supplemented during completion of the questionnaire. Data are directly stored electronically, avoiding unreadable handwriting and subsequent mistakes [26]. Data processing via Internet research saves time, especially in studies with many participants. Respondents are able to complete an Internet questionnaire at any time of day, anywhere.

We have shown that it is possible to detect patients with GERD symptoms through a dedicated website. This method can also be used for other conditions. We found that over 150,000 respondents completed the GerdQ questionnaire made accessible online on a health information website, emphasizing the need for disease information on the Internet. However, the skills of the general population to adequately seek health information on the Internet have been shown to be insufficient [27]. These deficiencies varied from problems with opening various common file formats and using hyperlinks embedded in different formats, to problems with appropriately evaluating the information they found [27].

In our study, only 10% of invited respondents completed the QOLRAD questionnaire. We consider the low response rate on completing the QOLRAD questionnaire to be the main drawback of research via an open access questionnaire. Respondents lack face-to-face contact and miss any relationship with the researchers, reducing their willingness to complete a questionnaire without any expected personal gain. A previous study by McCambridge et al assessed the effect of length and relevance of questionnaires on completion rates [28]. They found that only relevance, and not length of the questionnaire, influenced response rate. Another limitation of Internet research is that researchers are unaware of the accuracy of the given information. However, this also applies partly to telephone survey and paper-based questionnaires.

### Partial Responsiveness in Proton Pump Inhibitor Users

We used the GerdQ self-assessment questionnaire to identify partial responsiveness in PPI users. This is a novel and very promising feature of the GerdQ. We found that almost 90% of all PPI users had heartburn or regurgitation, sleep problems, or over-the-counter acid suppressive medication use for more than 1 day per week. Of the PPI users, 62% reported persistent symptoms on at least 4 days during the preceding week. Respondents with symptoms persisting at least 4 days per week reported the lowest health-related quality of life in our survey.

A recently published systematic review found that reflux symptoms during PPI therapy persisted in 17%-45% of patients in primary care and the general population [14]. We found a higher proportion of partial responders. This may be due to three independent elements. First, the definitions used in the included articles of the systematic review were not uniform and did not take aspects of quality of life into account. Second, in our study, all website visitors could complete the GerdQ, including those with comorbidity, who are normally excluded from trials. To obtain a maximal treatment effect in clinical trials, respondents with a high risk for decreased efficacy are normally excluded [29]. Third, people with incomplete symptom relief are likelier to search the Internet for more information.

### Strengths and Limitations

Our study has several strengths. We included over 130,000 participants in our study, which is the largest population studied for GERD so far [7,8,30]. We used a new, innovative way to collect data. Online data collection can be adequately used in the Netherlands, because more than 85% of Dutch inhabitants already had Internet access in 2008. This is the highest Internet coverage in Europe and would only have increased further during the last 4 years [31]. Using the GerdQ as a promising tool to assess the response of GERD patients to PPI therapy is a novelty. The GerdQ can be used as an easy and quick questionnaire to identify people with an incomplete response. Studies have demonstrated that most physicians presume that PPI therapy is effective in GERD [32]. However, PPIs do not help a significant percentage of patients, which is related to a decreased health-related quality of life [33,34].

Our study also has limitations. First, we have to take selection bias into account. Online health information seekers are probably younger and more educated than are people who search for health information offline [35]. We hypothesize that respondents with more severe symptoms might be overrepresented, as they are likely more motivated to search for information [36]. However, a US survey comparing characteristics of offline and online health information seekers found that online seekers reported a better health status [35]. Another aspect of selection bias in our study is that only a minority of respondents completed the QOLRAD questionnaire. A second limitation is that information regarding comorbidity, medical history, or use of other medications was not available. Third, respondents with suspected GERD symptoms did not undergo endoscopy or pH recording. However, previous research demonstrated that the GerdQ has the same sensitivity and specificity as a gastroenterologist in diagnosing GERD [18].

### Implications

The results of our study have some important implications for clinical practice. Many persons searching the Internet for information about reflux have GERD. This generates new opportunities for using the Internet to recognize and treat GERD. It is possible to detect people with GERD and to advise them at first to adjust their lifestyle and take an over-the-counter medication. If these measures are ineffective, these people can be advised to seek medical treatment. People can also regularly complete the GerdQ self-assessment questionnaire via the Internet to assess the effectiveness of their treatment. If they are dissatisfied, they can contact a health care practitioner.

Most PPI users searching the Internet report persistent symptoms or use over-the-counter medication in addition to PPI treatment. General practitioners and gastroenterologists assume that most patients with GERD are adequately treated [32], while our study showed the contrary. Health care providers can now use the GerdQ at every consultation to assess persistent symptoms on PPI therapy and the impact of reflux symptoms on daily life. When necessary, treatment can be adjusted. Further research should investigate the superiority of GerdQ-assisted practice over standard care. The first study to assess incorporation of the GerdQ in daily practice was recently published [37]. It compared the GerdQ with an endoscopy-based approach for diagnosis and initial treatment of GERD, and concluded that using the GerdQ reduced health care costs with comparable efficacy.

We have shown that it is feasible to find patients through a dedicated website for GERD. This concept will also be applicable to other conditions and diseases.

### Conclusions

The GerdQ self-assessment questionnaire was completed by over 130,000 website visitors. Two-thirds of respondents who did not use PPIs obtained a score suggestive of GERD. The prevalence of partial responsiveness to PPI therapy was high. Respondents reporting a high impact of GERD had a decreased disease-specific health-related quality of life. Identification of people with GERD through a GERD information website has been shown to be feasible.

## Multimedia Appendix 1

The GerdQ self-assessment questionnaire.

## Abbreviations

GERD: gastroesophageal reflux disease
PPI: proton pump inhibitor
QOLRAD: Quality of Life in Reflux and Dyspepsia
