# Supplementary material for: Open Access Capture of Patients With Gastroesophageal Reflux Disease Using an Online Patient-Reported Outcomes Instrument
Source: Interact J Med Res. 2012 Sep 26;1(2):e7. doi: 10.2196/ijmr.2101 (PMC3626138; doi:10.2196/ijmr.2101)
Supplement: Supplementary file 1 [file ijmr_v1i5e7_app1.pdf]

Multimedia appendix: The GerdQ self-assessment questionnaire

Symptoms present in the last 7 days

		Symptom presence		
		0 days	1 day	2[illegible]
	Question:			
1.	How often did you have a burning feeling behind your breastbone (heartburn)?	0	1	[illegible]
2.	How often did you have stomach contents (liquid or food) moving upwards to your throat or mouth (regurgitation)?	0	1	[illegible]
3.	How often did you have a pain in the center of the upper stomach?	3	2	[illegible]
4.	How often did you have nausea?	3	2	[illegible]
5.	How often did you have difficulty getting a good night's sleep because of your heartburn and/ or regurgitation?	0	1	[illegible]
6.	How often did you take additional medication for your heartburn and/ or regurgitation other than what the physician told you to take (such as Maalox)?	0	1	[illegible]

GerdQ symptom scores:
GerdQ < 8: low probability for GERD
GerdQ ≥ 8 and ≤ 3 on questions 5 and 6 (impact questions): GERD with low impact on daily life
GerdQ ≥ 8 and ≥ 3 on questions 5 and 6 (impact questions): GERD with high impact on daily life
